# Supplementary material for: A UMLS-based spell checker for natural language processing in vaccine safety
Source: BMC Med Inform Decis Mak. 2007 Feb 12;7:3. doi: 10.1186/1472-6947-7-3 (PMC1805499; doi:10.1186/1472-6947-7-3)
Supplement: Additional file 13 — RAP application directory. Sets up directory for RAP (RDF application for PHP) source code [file 1472-6947-7-3-S13.gz › rap/doc/phpdoc/default/RDF.html]

xml version="1.0" encoding="iso-8859-1"?


Docs For Class RDF


## Class RDF

Description

Description |
Methods (details)

Located in /vocabulary/RDF\_C.php (line 19)

Method Summary

Description |
Methods (details)

void
ABOUT
()

void
ABOUT\_EACH
()

void
ABOUT\_EACH\_PREFIX
()

void
ALT
()

void
BAG
()

void
BAG\_ID
()

void
DATATYPE
()

void
DESCRIPTION
()

void
FIRST
()

void
ID
()

void
LI
()

void
LITERAL
()

void
NIL
()

void
NODE\_ID
()

void
OBJECT
()

void
PARSE\_TYPE
()

void
PARSE\_TYPE\_RESOURCE
()

void
PREDICATE
()

void
PROPERTY
()

void
RDF\_LIST
()

void
RESOURCE
()

void
REST
()

void
SEE\_ALSO
()

void
SEQ
()

void
STATEMENT
()

void
SUBJECT
()

void
TYPE
()

Methods

Description |
Methods (details)

ABOUT (line 112)

void

ABOUT
()

ABOUT\_EACH (line 118)

void

ABOUT\_EACH
()

ABOUT\_EACH\_PREFIX (line 124)

void

ABOUT\_EACH\_PREFIX
()

ALT (line 22)

void

ALT
()

BAG (line 28)

void

BAG
()

BAG\_ID (line 130)

void

BAG\_ID
()

DATATYPE (line 172)

void

DATATYPE
()

DESCRIPTION (line 100)

void

DESCRIPTION
()

FIRST (line 76)

void

FIRST
()

ID (line 106)

void

ID
()

LI (line 160)

void

LI
()

LITERAL (line 148)

void

LITERAL
()

NIL (line 58)

void

NIL
()

NODE\_ID (line 166)

void

NODE\_ID
()

OBJECT (line 94)

void

OBJECT
()

PARSE\_TYPE (line 142)

void

PARSE\_TYPE
()

PARSE\_TYPE\_RESOURCE (line 154)

void

PARSE\_TYPE\_RESOURCE
()

PREDICATE (line 88)

void

PREDICATE
()

PROPERTY (line 34)

void

PROPERTY
()

RDF\_LIST (line 52)

void

RDF\_LIST
()

RESOURCE (line 136)

void

RESOURCE
()

REST (line 70)

void

REST
()

SEE\_ALSO (line 178)

void

SEE\_ALSO
()

SEQ (line 40)

void

SEQ
()

STATEMENT (line 46)

void

STATEMENT
()

SUBJECT (line 82)

void

SUBJECT
()

TYPE (line 64)

void

TYPE
()

Documentation generated on Fri, 17 Dec 2004 16:17:21 +0100 by phpDocumentor 1.3.0RC3
